# Amplification and bioinformatics analysis of conserved FAD-binding region of L-amino acid oxidase (*LAAO*) genes in gastropods compared to other organisms

**DOI:** 10.1016/j.csbj.2018.02.008

**Published:** 2018-03-02

**Authors:** Wipawadee Suwannapan, Pramote Chumnanpuen, Teerasak E-kobon

**Affiliations:** a Department of Genetics, Faculty of Science, Kasetsart University, Bangkok 10900, Thailand; b Department of Zoology, Faculty of Science, Kasetsart University, Bangkok 10900, Thailand; c Computational Biomodelling Laboratory for Agricultural Science and Technology, Kasetsart University, Bangkok 10900, Thailand

**Keywords:** L-amino acid oxidase (LAAO), FAD-binding region, Gastropods, Pattern analysis, Clustering

## Abstract

This study aimed to investigate the conserved FAD-binding region of the L-amino acid oxidase (*LAAO*) genes in twelve gastropod genera commonly found in Thailand compared to those in other organisms using molecular cloning, nucleotide sequencing and bioinformatics analysis. Genomic DNA of gastropods and other invertebrates was extracted and screened using primers specific to the conserved FAD-binding region of *LAAO*. The amplified 143-bp fragments were cloned and sequenced. The obtained nucleotide sequences of 21 samples were aligned and phylogenetically compared to the *LAAO*-conserved FAD-binding regions of 210 other organisms from the NCBI database. Translated amino acid sequences of these samples were used in phylogenetics and pattern analyses. The phylogenetic trees showed clear separation of the conserved regions in fungi, invertebrates, and vertebrates. Alignment of the conserved 47-amino-acid FAD-binding region of the LAAOs showed 150 unique sequences among the 231 samples and these patterns were different from those of other flavoproteins in the amine oxidase family. An amino acid pattern analysis of five sub-regions (bFAD, FAD, FAD-GG, GG, and aGG) within the FAD-binding sequence showed high variation at the FAD-GG sub-region. Pattern analysis of secondary structures indicated the aGG sub-region as having the highest structural variation. Cluster analysis of these patterns revealed two major clusters representing the mollusc clade and the vertebrate clade. Thus, molecular phylogenetics and pattern analyses of sequence and structural variations could reflect evolutionary relatedness and possible structural conservation to maintain specific function within the FAD-binding region of the LAAOs in gastropods compared to other organisms.

## 1 Introduction

Gastropods are the most diverse taxonomic group of molluscs living in a wide range of habitats, particularly in tropical and subtropical areas. The gastropods are generally classified into operculum-bearing prosobranchs, air-breathing pulmonates, and opisthobranchs (or sea slugs and sea hares). As Thailand is one of the biodiversity hotspots of the world and gastropods can live in diverse habitats, many gastropods are commonly found in Thailand, including pond snails (*Physa* sp., *Lymnaea* sp., *Biomphalaria* sp., *Bithynia* sp., *Viviparus* sp., *Pomacea canaliculata* and *Melanoides* sp.), garden snails (*Achatina fulica* and *Cryptozona siamensis)*, forest snails (*Cyclophorus volvulus)*, land slugs (*Semperula siamensis)*, sea snails (*Babylonia areolata*) and sea slugs (*Jorunna funebris*). Some of these are nutritious human foods (*Viviparus* sp., *B. areolata* and *C. volvulus)*, while other gastropods are harmful agricultural pests (*P. canaliculata*, *C. siamensis*, and *A. fulica*) and recognized as intermediate hosts of many nematode parasites in humans. Gastropods secrete mucus to facilitate their locomotion and hydrate their body surfaces. The mucus allows the snails to trail home, adhere to substrates and protect themselves from skin damage and microbial infection [1]. Gastropod mucus contains mostly water, electrolytes, minerals, glycoconjugates, proteoglycans, small peptides and glycoproteins including L-amino acid oxidases (LAAOs), a member of the amine oxidase family (PF01593) [[2], [3], [4], [5], [6], [7], [8]].

LAAO is a flavoenzyme that requires flavin adenine dinucleotide (FAD) or a quinone as a cofactor [9,10]. This enzyme acts as an innate immune defence by catalysing the oxidative deamination of an L-amino acid substrate to produce an alpha-keto acid, ammonia and hydrogen peroxide [11]. LAAO is also recognized as an important bioactive protein in defence against bacterial infections and cancers [[12], [13], [14], [15], [16]]. LAAOs are widely distributed in several organisms including bacteria, fungi, fish, snakes, and mammals as well as gastropods [11]. The protein structures of these enzymes and their related amine oxidases have been elucidated in some bacteria (*Streptomyces* sp. and *Streptococcus oligofermentans)* and reptiles (*Calloselasma rhodostoma*), showing three common domains: an FAD-binding domain, a substrate-binding domain, and a helical domain [17,18]. The FAD-binding domain consists of two conserved motifs, the FAD-binding motif (GxGxxG…hxhxE) and the GG motif (RxGGRxxS/T), where x indicates any amino acid residue and h indicates a hydrophobic residue [9]. The first and second glycine residues of the FAD-binding motif are important to the formation of the conserved structure of this motif, while the second glycine assists in making close contact with the phosphate group of the FAD molecule [19]. The first glycine of the GG motif is close to the FAD-binding motif, and the second residue interacts with the ribose molecule of the FAD cofactor [9].These conserved motifs form an initial beta-alpha-beta fold which is the most conserved part of the Rossmann fold structure of the LAAO proteins [20]. Variations within these two motifs have been previously reported, such as distinct conserved GG motifs in the LAAOs of *Streptococcus* spp. (RxGKK) and *Pseudoalteromonas* spp. (RxGGH) [21]. Variations within the non-conserved residues (x) of the FAD-binding motif were shown when comparing LAAOs from fish (*Sebastes schlegelii* and *Scomber japonicus*), a snake (*Pseudechis australis*) and gastropods (*Aplysia kurodai*, *Aplysia californica*, and *Achatina fulica*) [22], but a detailed analysis of these “x” residues has not been reported.

A previous phylogenetic analysis of 54 partial amino acid sequences inclusive of the FAD-binding region of LAAOs from bacteria and animals by Hughes [11] proposed the classification of LAAOs into two clusters. These two clusters were the mollusc-related subfamily and the vertebrate-related subfamily. The first subfamily included all LAAO sequences from a few gastropod species, three bacterial phyla (*Bacteroidetes*, *Firmicutes*, and *Proteobacteria*), and *Marseillevirus*. The second cluster included all sequences from vertebrates and three bacterial phyla (*Actinobacteria*, *Chloroflexi*, and *Firmicutes*). The occurrence of these two clusters suggested that the separation of the two subfamilies may have occurred when the two major clades of bacteria diverged, approximately three billion years ago [23]. Similarly, Campillo-Brocal et al. [10] reported that the phylogenetic relationships of LAAO enzymes from animals were separated into vertebrate-related and gastropod-related groups. These findings suggest the possible separate evolution of LAAO enzymes in the innate immune systems of both animal groups [11,24].

However, little information is known about the variation of LAAO enzymes in gastropods or in other invertebrates of the second cluster in the previous research due to limited number of the gastropod LAAO samples. Therefore, this study began investigating the FAD-binding region, which consists of the most conserved Rossmann fold of the *LAAO* genes in 12 gastropod species (representing prosobranch, pulmonate, and opisthobranch) together with nine other invertebrates commonly found in Thailand by gene amplification, molecular cloning, and nucleotide sequencing. The molecular relationships of this conserved region in gastropods with those in other organisms were analysed at the levels of nucleotide and amino acid sequences, and protein structures were compared by using phylogenetics and pattern analyses. The information of this conserved region would assist further investigation of sequences and structures of the gastropod LAAOs in more details.

## 2 Materials and methods

### 2.1 Selection and collection of gastropod and other invertebrate samples

Triplicate samples used in this study included 12 gastropod genera covering the three major groups (six pulmonates, six prosobranchs, and one opisthobranch), other classes of mollusks (five bivalves and one cephalopod considered as sister taxa) and other invertebrates in different phyla (two arthropods and one annelid) (see Supplementary Table 1). These commonly-found gastropod samples were collected from different habitats (lawns and gardens, freshwater ponds, forest, river and sea) in Thailand and were maintained in rearing chambers or preserved in 95% ethanol before the DNA extraction process. While the samples of commonly-found bivalves, cephalopods, and other invertebrates were selected and purchased from local fresh markets and preserved at −80 °C until use.

### 2.2 Amplification and sequencing of the conserved FAD-binding region in *LAAOs*

Genomic DNA was extracted using the GF-1 Nucleic Acid Extraction Kit (Vivantis Technologies, Oceanside, CA, USA). The FAD-binding and GG conserved motifs of the FAD-binding domain were amplified from the genomic DNAs using primers specific to the most conserved FAD-binding region (143-bp) of an *LAAO* gene, namely achacin in *A. fulica*: *LAAO2*-F (5′-TAGACGTTGCTGTGGTCGG-3′) and *LAAO2*-R (5′-GGGGACGTTAGGCAAGTG-3′). The achacin was the first full-length *LAAO* gene that was sequenced and characterized in gastropod [25]. PCR was conducted using 50 ng of the genomic DNA, 2 mM of the forward and reverse primers, 2 mM of dNTPs**, 10× Buffer A**, 50 mM MgCl_2_, and 5 U of Taq polymerase (Vivantis Technologies). Thermal cycling was performed with an initial denaturation step at 94 °C for 3 min, followed by 34 cycles of denaturation at 94 °C for 30 s, annealing at 55 °C for 30 s, extension at 72 °C for 1 min, and a final extension step of 72 °C for 5 min using an Eppendorf MasterCycler EP Gradient Thermal Cycler (Eppendorf, Hauppauge, NY, USA). PCR products were separated by 2% agarose gel electrophoresis and stained with 0.8% ethidium bromide before visualization under UV light using a Gel Doc™ XR+ system (Bio-Rad, Hercules, CA, USA). The PCR products were then cloned into the pGEM-T Easy vector (Promega, Madison, WI, USA). Ligation was conducted using 50 ng of the PCR products, 2× Rapid ligation buffer, 3 Weiss units/μl of ligase (Promega) and 50 ng of the vector. The ligation reaction was incubated overnight at 4 °C before mixing with 50 μl of *E*. *coli* DH5α competent cells and incubating on ice for 30 min. Transformation was conducted by heat-shocking at 42 °C for 1 min before placing on ice again for 2 min. Next, 400 μl of LB medium was added and the mixture was incubated in a shaker (Lab Companion, Shelburne, VT, USA) for 1 h at 37 °C and 180 rpm. Then, 300 μl of the transformation reaction was spread onto LA (Luria agar) plates containing 100 mg/mL of ampicillin (Vivantis Technologies), 0.1 M IPTG (Vivantis Technologies) and 20 mg/mL X-gal (Vivantis Technologies), and the plates were incubated overnight at 37 °C. White colonies were selected and checked by PCR amplification using the T7 (5′-TAATACGACTCACTATAGGG-3′) and SP6 (5′-TATTTAGGTGACACTATAG-3′) primers specific to the plasmid and the *LAAO2*-F and *LAAO2*-R primers. The transformed colonies harbouring the 143-bp products were picked and cultured in 5 mL of LB medium containing 100 mg/mL of ampicillin and incubated overnight on a shaker at 37 °C and 180 rpm. The recombinant plasmids were isolated from the cultured bacteria using BioFact™ Plasmid Mini Prep Kit (BioFact, Yuseong Gu, Daejeon, Korea). The nucleotide sequences of the conserved motifs were amplified and sequenced (Macrogen, Geumcheon-gu, Seoul, Korea).

### 2.3 Phylogenetic analyses of nucleotide and amino acid sequences of the conserved FAD-binding region of *LAAOs* in gastropods and other organisms

The newly obtained nucleotide sequences of the conserved FAD-binding sequence of *LAAO*s from gastropods and other invertebrates were aligned, edited and compared with those of other organisms downloaded from the National Center for Biotechnology Information (NCBI) database using MAFFT version 7 [26]. Two-hundred and six *LAAO* sequences obtained from the NCBI database were from eukaryotes, while four sequences were from the *LAAO* and its distantly-related genes in bacteria. Sequence trimming and editing was performed by BioEdit [27]. A nucleotide phylogenetic tree of the conserved FAD-binding region of *LAAO* was constructed based on the Kimura 2-parameter +Gamma (G) + Invariable site (I) model using the maximum likelihood method as an optimal model [28]. The trees were assessed by 1000 bootstrap replicates [29] using MEGA version 6.0 and visualized with iTOL [30]. Then, the nucleotide sequences were translated into amino acid sequences by Expasy (http://web.expasy.org/translate/). The protein phylogenetic tree was constructed from the conserved amino acid sequences of the FAD-binding region using the maximum likelihood method based on the LG + Gamma (G) model [31]. The trees were assessed and visualized as with the nucleotide phylogenetic trees mentioned earlier.

### 2.4 Amino acid pattern analysis of the conserved FAD-binding region in LAAOs of gastropods compared to those of other organisms

To gain further understanding of molecular evolution within the conserved FAD-binding regions of LAAOs, the protein sequence alignment from Section 2.3 was divided into five sub-regions based on the conserved residues of the FAD-binding and GG motifs: 1) five amino acids before FAD-binding motif (bFAD), 2) six amino acids of the FAD-binding motif (FAD) containing the conserved GxGxxG residues, 3) 21 amino acids between the FAD-binding and GG motifs (FAD-GG), 4) eight amino acids of the GG motif (GG), and 5) seven amino acids after the GG motif (aGG). The aligned sequences of each sub-region were analysed as follows: i) pattern identification based on amino acid order and biochemical properties within the sub-region; ii) combining patterns from these five sub-regions into a set of five digits (sub-regions 1, 2, 3, 4, and 5); iii) hierarchical clustering of the five-digit patterns and constructing dendrograms by using R scripts; and iv) comparison of the patterns of these five sub-regions to the patterns derived from the full sequences of the conserved FAD-binding region in the LAAO proteins and to the patterns derived from other 18,241 flavoproteins in the amino oxidase protein family (PF01593) obtained from the Pfam database (https://pfam.xfam.org/) by using R scripts. Amino acid variation within the conserved FAD-binding region and sub-regions was analysed and presented as an alphabetical plot using the LogoBar program [32].

### 2.5 Structural pattern analysis of the conserved FAD-binding region in LAAOs of gastropods compared to those of other organisms

Structures of amino acid patterns from Section 2.4 were predicted by the SWISS-MODEL program [33] based on automated template selection. The predicted protein structures were quality checked using PROCHECK [34]. The predicted protein structures were aligned by PDBeFold [35] and visualized by RasWin [36]. Secondary structural patterns of the conserved FAD-binding region of LAAOs were analysed by simplifying structural alignment results to numeric patterns (0–4) after residue-by-residue comparison of the PDBeFold results. The conserved residue contributing most to the conserved secondary structure obtained the highest score, while the residue contributing the least conserved structure received the lowest one. Zero indicated a residue in a highly structurally variable position, while four indicated a residue in the most highly structurally conserved region. These numeric patterns were clustered, and the dendrogram was drawn by using R scripts.

## 3 Results

### 3.1 Phylogenetic analyses of the conserved FAD-binding region in *LAAO*s of gastropods and other organisms

**Fig. 1:**
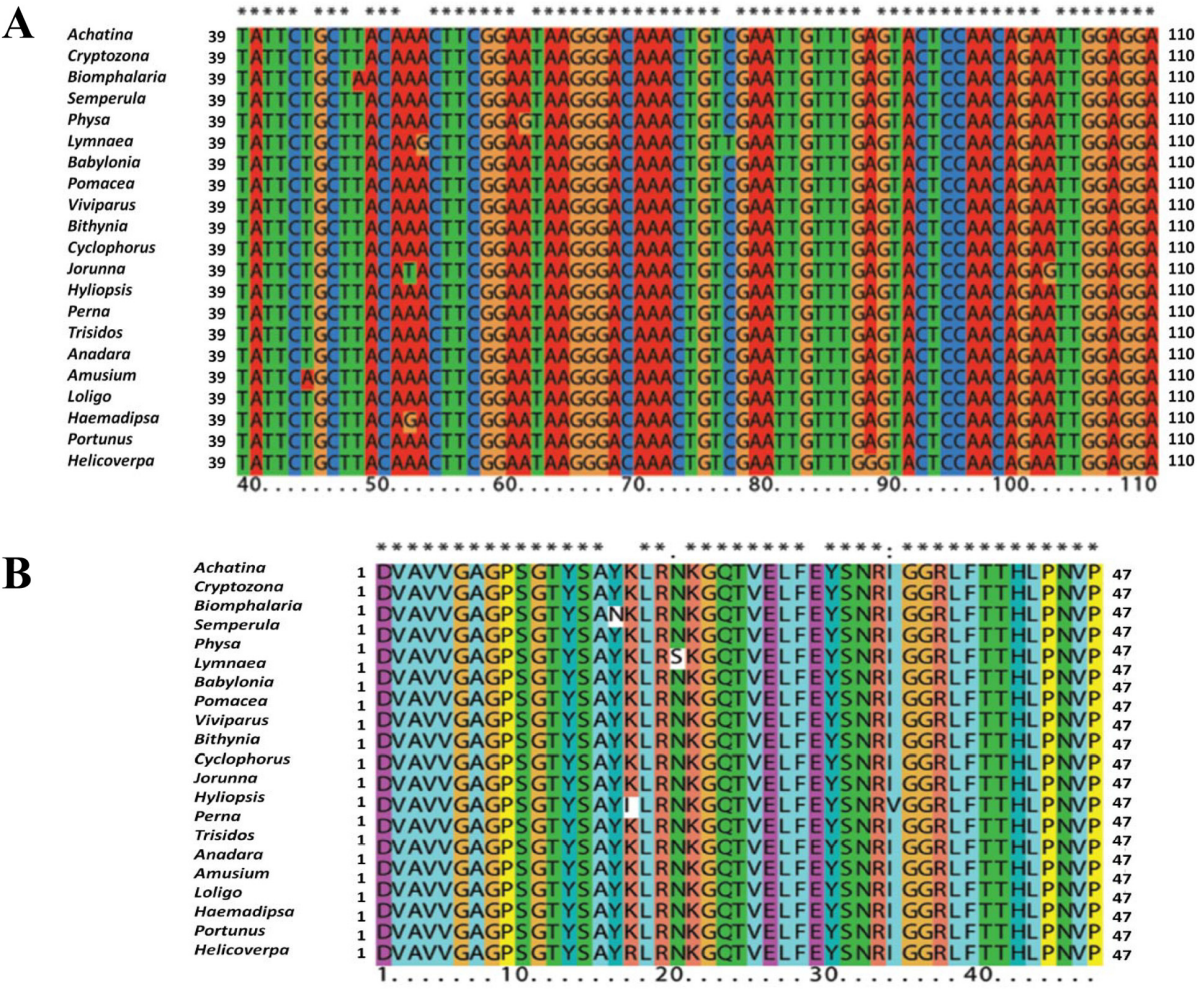
Multiple sequence alignment of nucleotide (A) and protein (B) sequences of the conserved FAD-binding region in the *LAAO* gene from twelve gastropods, six mollusc phyla from two sister taxa (five bivalves and one cephalopod), and four other invertebrates. Asterisks (*) indicate the conserved residues for both nucleotide and protein sequence alignments. For the nucleotide sequence alignment (A), four nucleotides are shown in different colours. For the protein sequence alignment (B), different colour labels represent different groups of amino acids: purple for D and E; blue for Y and H; yellow for P; green for S, T, Q, and N; orange for G; pink for K and R; cyan for V, A, L, F, and I; and grey for variable residues.

This study successfully amplified 143 bp of the conserved FAD-binding region of *LAAO* genes in twelve gastropods and nine other invertebrate samples by using our designed FAD-binding region-specific primer pairs. Blasting these sequences against a nucleotide sequence database confirmed the correct match to the FAD-binding region of the *LAAO* gene (achacin) of *A*. *fulica*. Multiple alignment of these 21 nucleotide sequences showed nucleotide substitutions at single variable residues in *Biomphalaria* sp. (position 48, T/A), *Physa* sp. (position 61, A/G), *A. pleurohectaus* (position 44, T/A), *Haemadipsa sylvestris* (position 52, A/C), and *Helicoverpa armigera* (position 88, A/G), and at two variable residues in *Lymnaea* sp. (position 53, A/G and position 77, C/T) and *J. funebris* (position 52, A/T and position 102, A/G) (Fig. 1A). Alignment of the translated protein sequences (47 amino acids) mostly showed missense mutations, including position 16 (Y/N) in *Biomphalaria* sp., position 20 (N/S) in *Physa* sp., position 17 (K/R) in *Haemadipsa sylvestris*, position 29 (E/G) in *Helicoverpa armigera*, and positions 17 (K/I) and 34 (I/V) in *J. funebris* (Fig. 1B). Silent mutations were also observed in *Lymnaea* sp. and *A. pleurohectaus*.

**Fig. 2:**
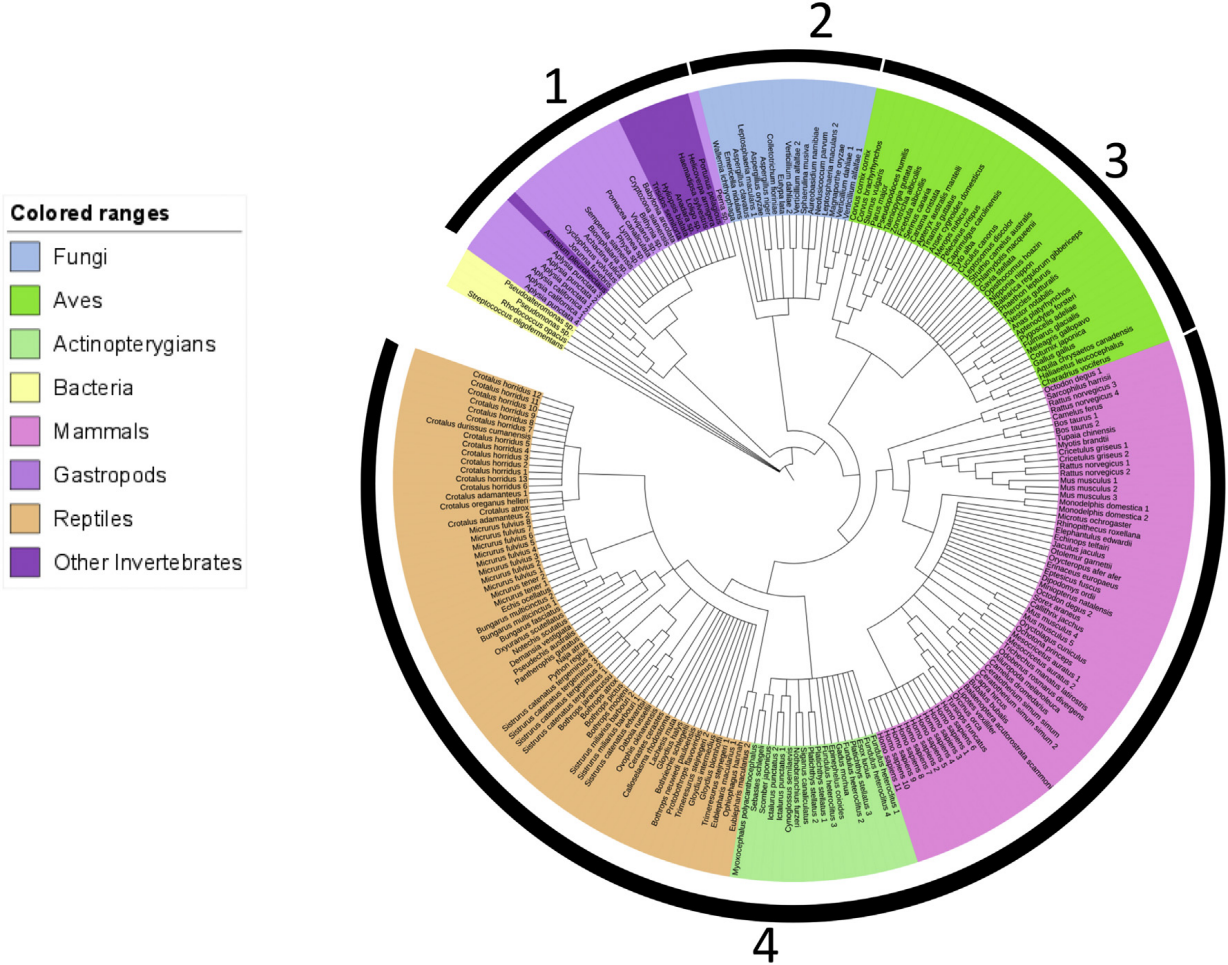
Nucleotide phylogenetic tree of 231 conserved FAD-binding sequences of the *LAAO* gene based on maximum likelihood with the Kimura 2-parameter model and 1000 bootstrap replicates. The samples are highlighted in different colours for bacteria, fungi, gastropods and other invertebrates, actinopterygians, Aves, reptiles, and mammals. Numbers over the black bars represent the clusters.

**Fig. 3:**
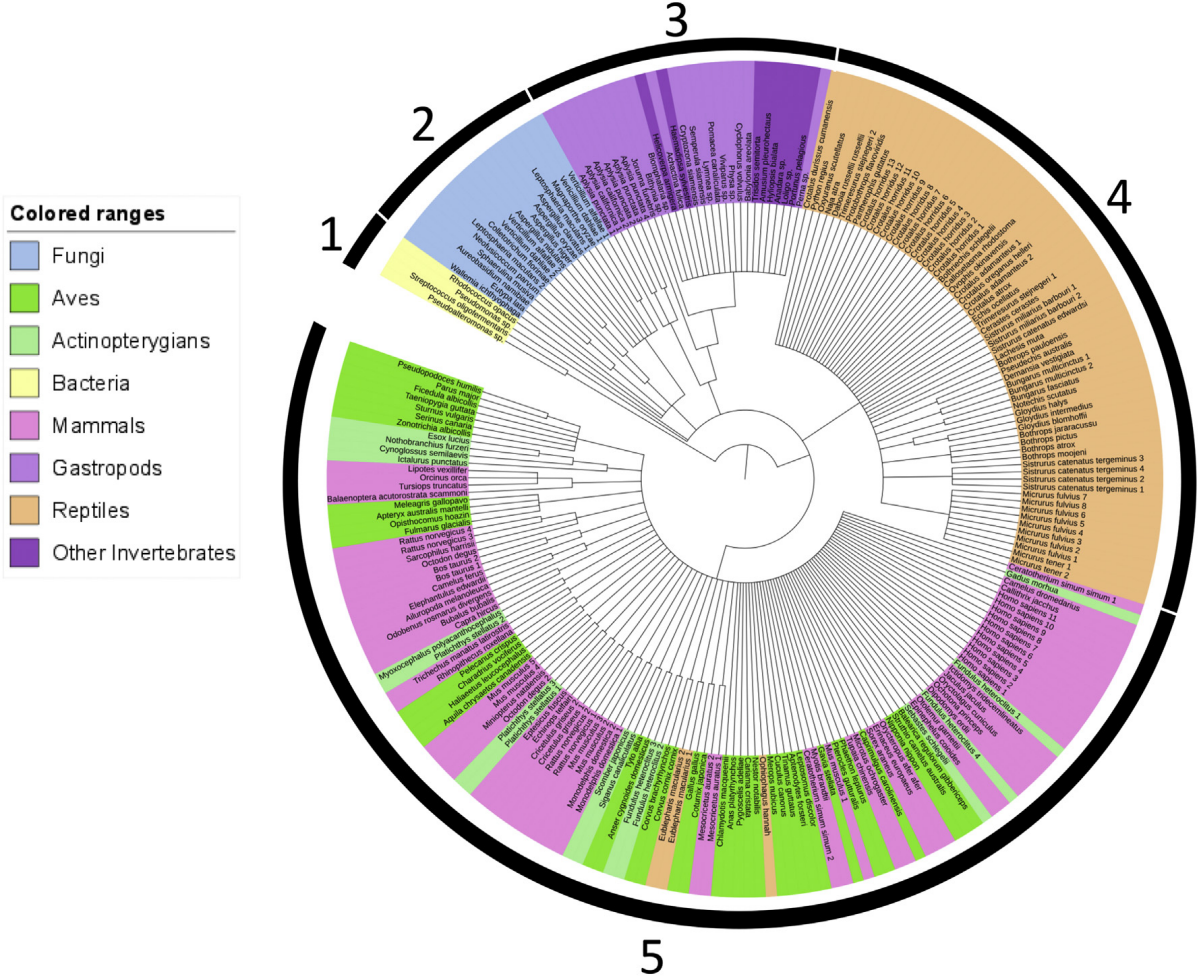
Protein phylogenetic tree of 231 conserved FAD-binding region in the LAAO proteins based on maximum likelihood with the Kimura 2-parameter model and 1000 bootstrap replicates. The samples are highlighted in different colours for bacteria, fungi, gastropods and other invertebrates, actinopterygians, Aves, reptiles, and mammals. Numbers over the black bars represent the clusters.

The LAAO conserved nucleotide and protein sequences of the FAD-binding region of these 21 samples were compared with those of other organisms from the NCBI database. The nucleotide phylogenetic tree of this 143-bp region was similar to the protein phylogenetic tree of the 47-amino-acid FAD-binding region of the LAAO proteins (Fig. 2, Fig. 3). The nucleotide tree showed separation of the bacterial LAAO-related conserved regions from those of other eukaryotic organisms (Fig. 2), while the protein tree grouped the sequences of *Pseudoalteromonas* sp. and *Streptococcus oligofermentans* with the eukaryotic cluster and those of *Rhodococcus opacus* and *Pseudomonas* sp. in separate branches (Fig. 3). The conserved sequences of gastropods and other invertebrates were grouped in the same cluster in both the nucleotide and protein trees. The conserved FAD-binding region in the *LAAO* of fungi was close to those of vertebrates, but this was not clearly shown in the protein tree (Fig. 2). The conserved regions of mammals, actinopterygians and reptiles were grouped together in the fourth cluster of the nucleotide tree, separate from the third group, which represented Aves (Fig. 2). However, the protein sequences of Aves, mammals, and a few reptiles (*Eublepharis macularius* and *Ophiophagus hannah*) were grouped with actinopterygians, separate from those of most reptiles (Fig. 3).

### 3.2 Amino acid patterns of the conserved FAD-binding region in LAAOs of gastropods compared to those of other organisms

**Fig. 4:**
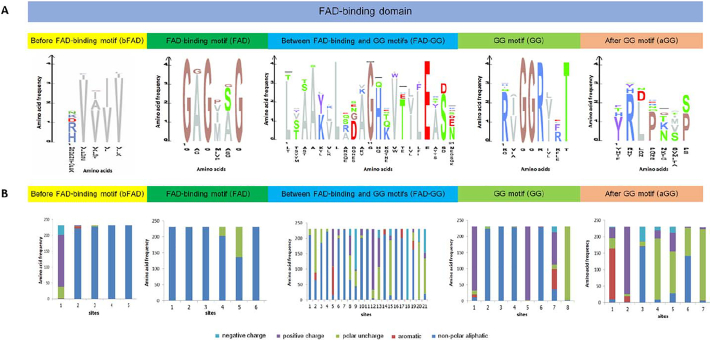
Amino acid frequencies and properties within 150 patterns of the conserved FAD-binding region and sub-regions of the LAAOs from 231 samples of gastropods and other organisms. Logo bar plots (A) represent the frequency of amino acids among the 150 patterns of the conserved region. This focussing region was subdivided into five sub-regions: before FAD-binding motif (bFAD), FAD-binding motif (FAD), between FAD-binding and GG motifs (FAD-GG), GG-motif (GG), and after-GG-motif (aGG) sub-regions. Different patterns within each sub-region were observed. The X axis of the plot represents amino acid positions, while the height of each letter shows the frequency of a particular amino acid at the same position across all observed patterns. Letters in the logo plot are one-letter amino acid codes. Bar plots (B) represent the frequency of amino acid properties within these five sub-regions. The X axis of the bar plot represents the amino acid positions within each sub-region, while the bar height shows the proportional frequency of amino acid properties at that position across all observed residues. The colours of the bars show different biochemical properties: blue for non-polar aliphatic amino acids; light blue for negatively charged amino acids; violet for positively charged amino acids; green for uncharged polar amino acids; and red for aromatic amino acids.

Analysis of the multiply aligned 47-amino-acid sequences of the FAD-binding region in the LAAOs from 231 samples (21 sequences from this study and 210 sequences from the database) showed 150 different amino acid patterns. These 150 patterns were further divided into five sub-regions (Fig. 4): 48 unique patterns of the before-FAD-binding-motif (bFAD, five amino acids) sub-region, 17 patterns of the FAD-binding-motif (FAD, six amino acids) sub-region, 117 patterns of the sub-region between the FAD-binding and GG motifs (FAD-GG, 21 amino acids), 54 patterns of the GG-motif (GG, eight amino acids) sub-region, and 83 patterns of the after-GG-motif (aGG, seven amino acids) sub-region. Amino acid sequences of these 150 patterns and their sub-regions were shown in Supplementary Fig. 1., Supplementary Fig. 2., Supplementary Fig. 3., Supplementary Fig. 4., Supplementary Fig. 5., Supplementary Fig. 6.. The FAD-GG sub-region had the highest number of patterns, followed by the aGG, GG, and bFAD sub-regions, while the FAD sub-region had the lowest number of patterns. Most of the patterns within these five sub-regions were unique to organismal groups, except in the FAD sub-region. The patterns of the FAD-binding region of the LAAO and its distantly-related gene in bacteria were clearly separate from the conserved region of LAAOs in other organisms.

Comparing all 48 patterns (five amino acids) of the bFAD sub-region (see Supplementary Fig. 2) showed high amino acid variation at the first position of this sub-region (Fig. 4A). Most amino acids in the first position were positively charged amino acids. Other positions mostly had non-polar aliphatic amino acids (valine, isoleucine, glycine, and methionine) (Fig. 4B). Half of the patterns in the FAD sub-region (see Supplementary Fig. 3) were mixed between different organisms. These first two sub-regions in the bacterial samples formed separate patterns to other organisms. Comparing these 17 patterns of the FAD sub-region showed three conserved glycine positions (GxGxxG) in all patterns (Fig. 4A), where x represents any amino acid residue. The second and fifth positions had similar variations of alanine, glycine, and serine, while the fourth position showed the highest variation. This FAD sub-region contains mostly non-polar aliphatic amino acids such as glycine and alanine (Fig. 4B), similar to those of the bFAD sub-region.

For the FAD-GG sub-region (see Supplementary Fig. 4), 117 patterns were well classified according to organismal groups. A conserved lysine residue at position 7 appeared in almost all patterns, excluding only pattern 36 in reptiles and pattern 110 in bacteria. A conserved glycine (G) residue at position 11 was observed in all patterns, except in those of bacteria. A conserved glutamic acid (E) at position 18 was observed in almost all patterns, except pattern 108 in bacteria and pattern 116 in invertebrates (Fig. 4A). Deletions were observed within this sub-region in the bacterial samples (see Supplementary Fig. S4). Thus, the three conserved residues of the FAD-GG sub-region [L–x(3)–G–x(7)–E] are clearly shown in Fig. 4A; the number within the parentheses indicates the number of repeated residues. Comparing amino acid properties in Fig. 4B shows another conserved residue at position 16, which had only non-polar amino acid residues (L, V, and I), while other positions in this sub-region were variable. Thus, the conserved pattern within this sub-region could be further summarized as [L–x(3)–G–x(5)–[ILV]–x–E].

Most patterns in the GG sub-region were classified into different organismal groups (see Supplementary Fig. 5). Comparing the 54 patterns of the GG sub-region showed three conserved residues (two glycines (GG) at the third and fourth positions, arginine (R) at the fifth position, and threonine (T) at the eighth position) in all patterns, except patterns 51–53 in bacteria (Fig. 4A). The conserved residues in this sub-region could be summarized as [GGR–x(2)–T]. The amino acid properties within this sub-region were highly conserved, except in the seventh position. The pattern of [positively charged residue–non-polar residues (3)–positively charged residue–non-polar residue–x–uncharged polar residue] was also clearly observed (Fig. 4B).

The after-GG (aGG) sub-region was well separated by organism classification. Only pattern 45 and pattern 48 were shared between reptiles and actinopterygians (see Supplementary Fig. 6). Comparing 83 patterns of the aGG sub-region showed high variation, similar to that of the FAD-GG sub-region (Fig. 4A). Arginine (R) was dominant in the second position, while leucine (L), proline (P) and proline/serine (S) were prevalent in the third, fourth, and seventh positions. The seventh position had an uncharged polar residue, while other positions showed variation in chemical properties (Fig. 4B). No clear patterns could be summarized in this sub-region.

**Fig. 5:**
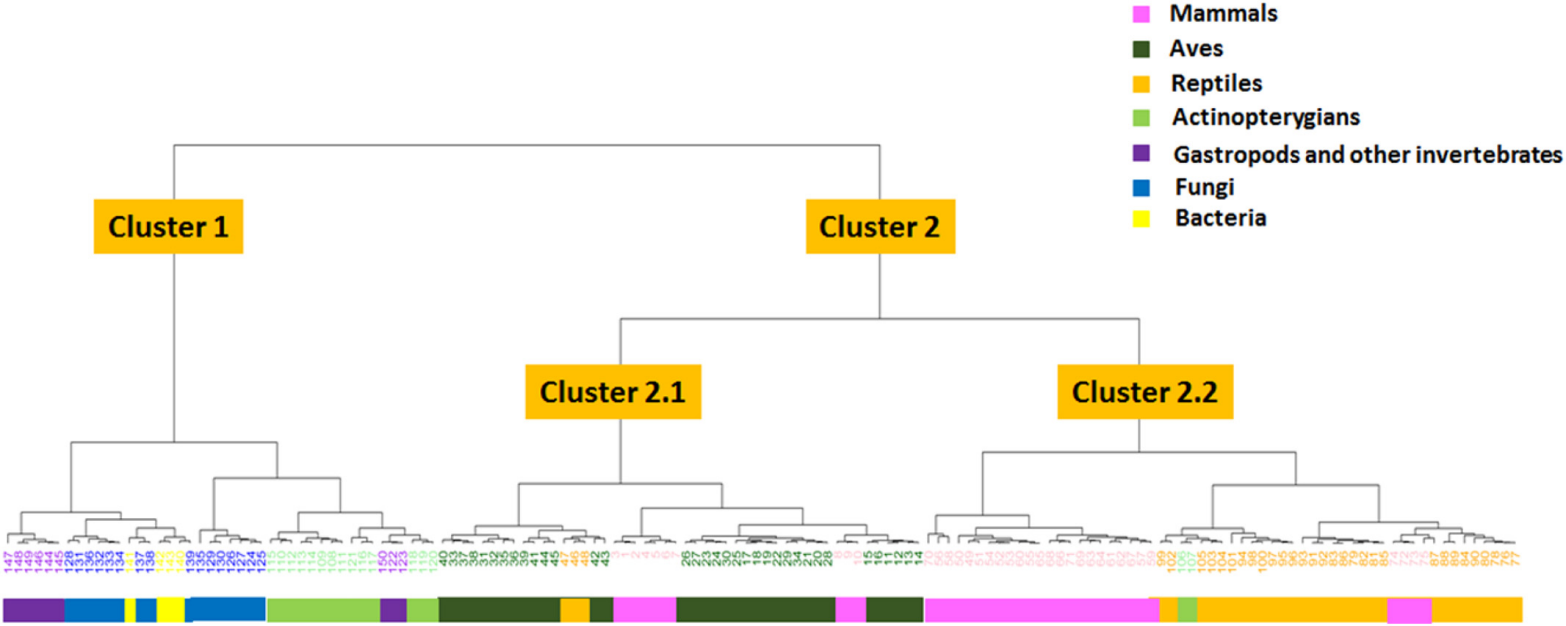
Dendrogram representing the clustering of 150 patterns of the conserved FAD-binding region of the LAAOs (47 amino acids) from 231 samples based on the patterns of the five sub-regions (bFAD, FAD, FAD-GG, GG, and aGG). Leaf numbers indicate the pattern numbers, which correlate with the pattern numbers in Supplementary Fig. 1. Colours show different organismal groups. Dendrogram representing the clustering of 150 patterns of the conserved FAD-binding region of the LAAOs (47 amino acids) from 231 samples based on the patterns of the five sub-regions (bFAD, FAD, FAD-GG, GG, and aGG). Leaf numbers indicate the pattern numbers, which correlate with the pattern numbers in Supplementary Fig. 1. Colours show different organismal groups.

Analysis of these five sub-regions yielded numeric patterns of five numbers for each sample representing the patterns of the bFAD, FAD, FAD-GG, GG, and aGG sub-regions. The patterns of these five numbers represented distinct characteristics of the 150 patterns of the conserved FAD-binding region in the LAAO proteins. Cluster analysis of these numeric patterns showed division of the 150 patterns of the conserved region into two major clusters (Fig. 5). The first cluster of 43 patterns belonged to the patterns of gastropods and other invertebrates (nine patterns), actinopterygians (14 patterns), bacteria (four patterns), and fungi (16 patterns). A relationship of the FAD-binding region in the fungal LAAOs and those of bacteria was also shown in this cluster. The second cluster included 107 patterns of all vertebrates and was further divided into two sub-clusters, 2.1 and 2.2. Cluster 2.1 included most Aves and some mammals and reptiles. The rest of the mammals and reptiles, along with a few actinopterygians, were in cluster 2.2. Thus, analysis of these amino acid patterns showed a close relationship of the FAD-binding region conserved in LAAOs among invertebrates and fungi which clearly separated from those of vertebrates. Similar scheme of the pattern analysis was also applied to 18,241 flavoproteins in the amine oxidase family. Results showed the overall of 16,365 unique patterns. Three-hundred and thirty-nine patterns of these were from the FAD-binding regions of the LAAOs including our 150 patterns which have not been completely deposited in the Pfam database.

### 3.3 Structural prediction of the conserved FAD-binding region in LAAOs of gastropods and other organisms

**Fig. 6:**
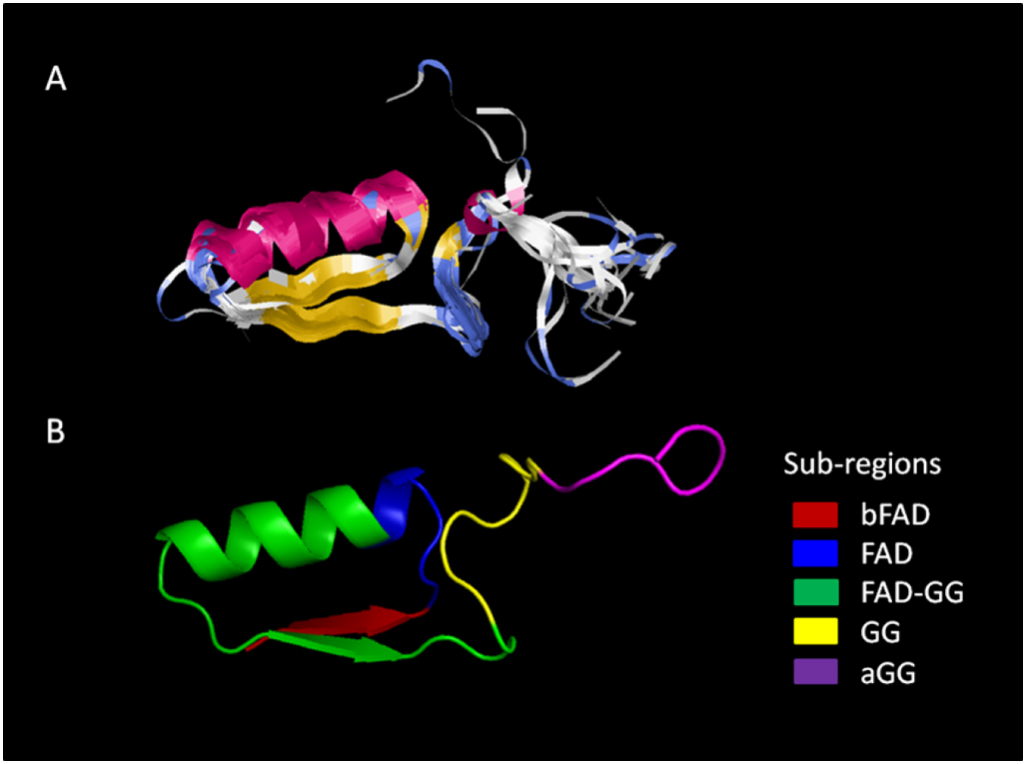
Predicted and superimposed structures of 150 amino acid patterns of the conserved FAD-binding region of LAAOs in gastropods and other organisms. The structures of 150 patterns were predicted and superimposed (A). Pink represents the alpha-helix, yellow represents the beta-sheet, and blue represents the random coiled-coil structures. Five sub-regions within this domain were distinctly coloured in (B).

Structures of the conserved FAD-binding region in LAAOs from 21 samples of gastropods and other invertebrates were predicted, and no significant difference was found. The amino acid variations within the conserved region of these 21 samples did not affect the structural configuration. The predicted FAD-binding region of these LAAOs had a beta-alpha-beta (βαβ) structural fold which was an important feature of the Rossmann fold (Fig. 6). The FAD-binding motif (FAD) was between the first beta-sheet (bFAD) and the middle alpha-helix (Fig. 6). The alpha-helix and the second beta-sheet were part of the FAD-GG sub-region, while the GG and aGG sub-regions constituted the coiled-coil structure after the second beta-sheet. The predicted structures of the prior 150 patterns were aligned and superimposed (Fig. 6). Clearly, the βαβ folds of these 150 structures were highly conserved, while the coiled-coil structures of the FAD and aGG sub-regions were quite variable. The secondary structure alignment of these 150 patterns showed three patterns (patterns 11, 117, and 141) that did not have similar conserved βαβ folds to the other patterns. Patterns 11 and 117 in the Aves and actinopterygians were predicted to have only a single conserved alpha-helix, whereas pattern 141 in bacteria was the most distinct structure which had an additional alpha-helical strand after the second beta-sheet.

**Fig. 7:**
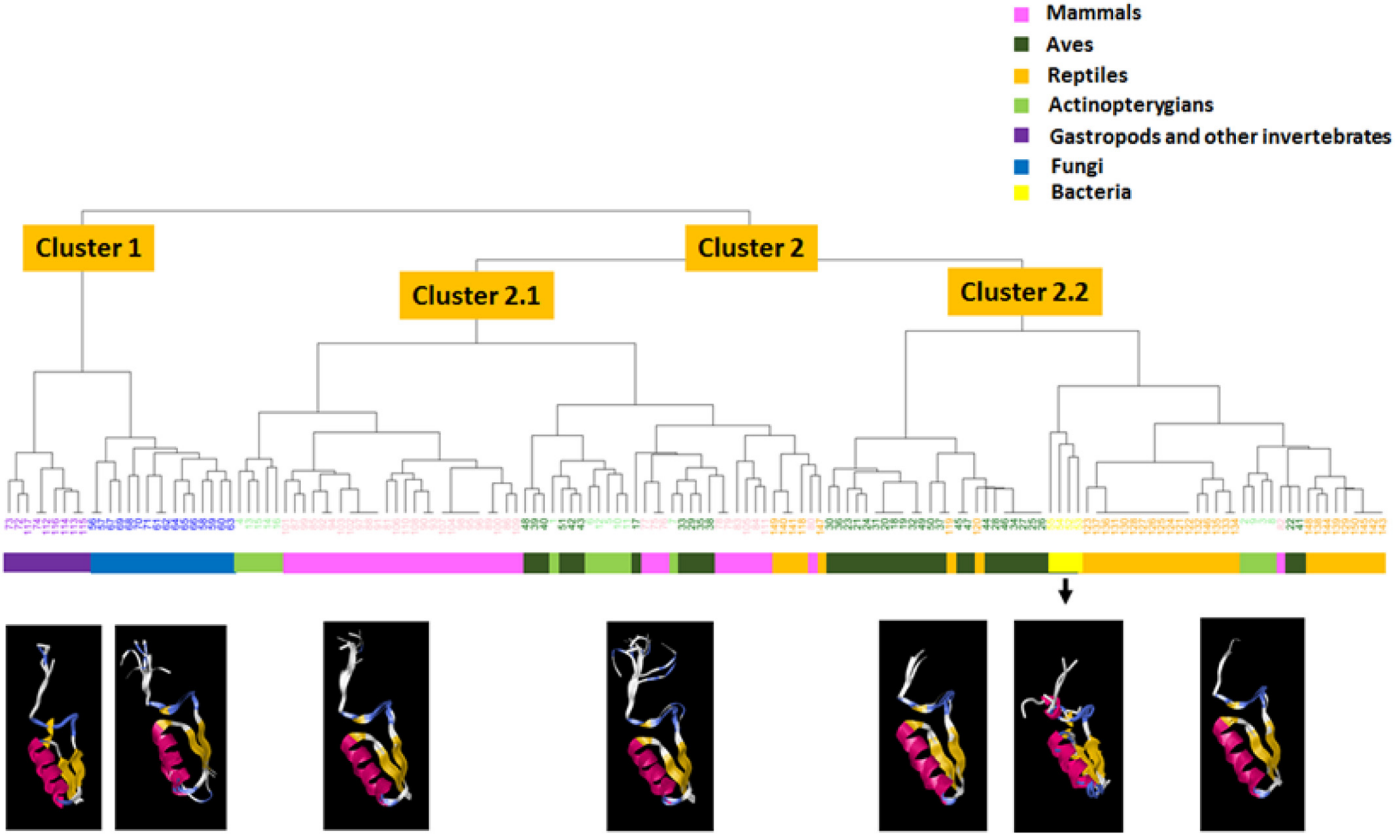
Clustering of 150 structural patterns of the conserved FAD-binding region of the LAAO proteins from gastropods and other organisms. These 150 patterns were decomposed from amino acid and secondary structure information into combinatorial patterns of five digits (0 to 4). Superimposed structures of the patterns within each cluster are shown below the cluster group.

Residue-by-residue mapping in relation to the secondary structures of these 150 structures decomposed their secondary structures into combinatorial patterns of five digits. The cluster analysis of these structural patterns showed division of these 150 patterns of the conserved FAD-binding region in LAAOs into two major clusters (Fig. 7). The first cluster contained the patterns of gastropods and other invertebrates. The second cluster included all vertebrates and bacteria, which were previously grouped within the first cluster in Fig. 5. The second cluster was further divided into two sub-clusters, 2.1 and 2.2. Cluster 2.1 included mammals, some actinopterygians, reptiles, and Aves. The rest were grouped with the patterns of bacteria in cluster 2.2. This analysis similarly showed a close relationship of the FAD-binding region between the LAAOs of invertebrates and fungi. The predicted structures of the patterns within each group of organisms were similar, particularly in the first four sub-regions including the βαβ folds (Fig. 7). The fifth sub-region (aGG) tended to vary across different groups. This sub-region in bacteria showed high variation and clearly separated from the mixed clade of vertebrates.

Comparison of the structural pattern analysis with the amino acid patterns of these 150 patterns of the FAD-binding region of the LAAOs in seven groups of organisms showed a clear separation between this region in the vertebrates and in the invertebrates and fungi (Fig. 6, Fig. 7). Similar numbers of clusters and sub-clusters were obtained. The dendrogram of amino acid patterns showed most mammals and reptiles grouped in sub-cluster 2.2, separated from the Aves in sub-cluster 2.1, while in the dendrogram of the structural patterns, most reptiles and Aves were in the same sub-cluster, separated from mammals, which were within another clade of mixed vertebrates. Another interesting observation was that bacteria and actinopterygians were in the first cluster in the dendrogram of the amino acid patterns, while these were grouped differently in the second cluster of the structural pattern dendrogram. However, both dendrograms consistently showed a close relationship of this conserved region among gastropods, other invertebrates, and fungi.

## 4 Discussion

This study examined the conserved FAD-binding region in *LAAO* genes of gastropods and other organisms. The *LAAO2* primer pairs showed specific amplification of the conserved region of the *LAAO* gene in all 21 samples from gastropods and other invertebrates, suggesting that this region could be highly conserved in invertebrates. This study was also the first report of the wide screening of this conserved FAD-binding region in several groups of gastropod and other invertebrate species. Nucleotide sequences were analysed by several approaches to understand the molecular evolutionary process of this region from the level of nucleotide sequences to amino acid sequences and protein structures.

The 143-bp nucleotide sequences of the conserved FAD-binding region in the *LAAO* gene of gastropods and other invertebrates in this study were highly conserved, with a few nucleotide variations causing changes in the protein sequences. Almost all the conserved regions of gastropods and other invertebrates in this study formed a monophyletic group, excluding sequences from an opisthobranch (*Jorunna funebris*) and a leech (*Haemadipsa sylvestris*). Compared to the conserved regions of other organisms in the NCBI database, this region in gastropods and other vertebrates clearly forms a monophyletic group at both nucleotide and amino acid sequence levels (Fig. 2, Fig. 3). In this study, the conserved regions of opisthobranchs (*Jorunna* and *Aplysia*) were shown to be different from those of other gastropods (pulmonates and prosobranchs); in fact, this region in the pulmonates and prosobranchs was even closer to those of other invertebrates (bivalve, cephalopod, and insect) than those of the opisthobranchs. This result emphasized the molecular evolutionary complexity and diversity of genes within the gastropods, which are the second largest group of animals, but surprisingly showed a few variations in this region compared to those of other organisms. A recent study by [37] on the evolution of gastropods based on mitochondrial genome comparison showed an interesting discrepancy between morphology-based and nucleotide-sequence-based phylogenetics. The morphological data indicated a close relationship between pulmonates and opisthobranchs within their own monophyletic groups, separate from the prosobranchs or caenogastropods. The nucleotide-based phylogeny conversely showed that the pulmonates and opisthobranchs were mixed and did not form separate monophyletic groups, although the separation of these groups from the prosobranchs and cephalopods remained clear. Our findings might not fully agree with the evolutionary history of these organisms, but these results highlighted the fact that the evolution of genes or sub-regions within genes could differ from the evolutionary pattern that based on the whole genes or whole organisms. This concept could also be supported by the evolutionary analysis of genes and genomes from 12 *Drosophila* species [38]. Although most genome features were similar in the flies, the authors found that genes in the defence response and proteolysis families had high rates of protein evolution and positive selection compared to other families. Hence, our results suggested that strong positive selection has protected the FAD-binding region within the *LAAO* gene of gastropods and other invertebrates in this study to achieve the co-factor binding role in the LAAO proteins, while the other parts of the LAAO proteins could be extremely diverse.

Our phylogenetic results were consistent with those of a previous study, which constructed phylogenetic trees from longer 218-amino-acid sequences of the LAAO proteins from viruses, bacteria, molluscs, and vertebrates [11]. Their phylogenetic trees showed a separation of these LAAO proteins into two major clades of vertebrates mixed with terrabacteria (*Actinobacteria*, *Chloroflexi*, and *Firmicutes*), or the vertebrate-related group, and the others that were grouped with hydrobacteria (*Bacteroides*, *Firmicutes*, and *Proteobacteria*), or the mollusc-related group. Hughes [11] also proposed that the divergence of these two groups could have occurred by duplication of this gene before the most recent common ancestor of bacteria and eukaryotes separated. Then, each clade might have lost one copy during adaptation and evolution within the lineage. Evidence of bacterial LAAOs clustered with both clades suggested that the gene duplication event could have occurred before these groups of bacteria diverged three billion years ago. Later, the *LAAO g*ene might have been horizontally transferred from terrabacteria to the vertebrate lineage, and from hydrobacteria to the invertebrate lineage, independently. Hughes [11] also reported the loss of *LAAO* genes in insects, and no *LAAO* genes from insects were found in the NCBI database. However, Kotaka [39] reported finding LAAOs in silkworm eggs (*Bombyx mori* L.), and this study also found the conserved domain of an *LAAO* gene in an insect sample. These results suggested that *LAAO* genes might remain present in some groups of insects. Thus, the present study successfully employed 47 amino acid residues of the conserved FAD-binding region in the LAAO proteins to understand the phylogenetic relationships of this region in a broader range of organisms, which suggested that the evolution of this gene could be driven by the maintenance of functional FAD-binding conserved region. Whereas, the rest of the LAAO proteins might diversify to different structures and activities. The information of these evolutionary changes could remain captured within their FAD-binding regions. Our study thus used this information to examine evolutionary story of this LAAO-conserved region in gastropods which were little known. The strong selection of the conserved region in gastropods and other invertebrates was intriguing, despite their high level of genetic diversity compared to other organisms.

This study designed pattern-analytical approaches to incorporate protein sequence and structure information along with phylogenetic data to understand molecular variation within the FAD-binding region of the LAAO proteins. Analysis of amino acid patterns in the five sub-regions found a lower number of patterns in the FAD and GG motifs than in the bFAD, FAD-GG, and aGG sub-regions, suggesting strong selection particularly on the sub-regions related to the function of the FAD-binding motifs and more relaxed constraint on the other three sub-regions (Fig. 4). The pattern analysis invented in this study was able to simplify the complex pattern of 47 amino acids of the conserved FAD-binding region into a pattern of five digits per sample. The pattern numbers in gastropods and other invertebrates were considerably lower than those of vertebrates, indicating possible stronger positive selection on this region of the invertebrates. This study found that the conserved amino acid pattern of the bFAD sub-region and the FAD motif was GxGxxG, as previously described by Dym [19]. Similarly, the GG motif was conserved as RxGGRxxT. The FAD-GG sub-region was the most variable, but some conserved residues were also observed as L-x(3)-G-x(5)-[ILV]-x-E in this study. Previous research showed that the last glutamate (E) was a signature residue for the FAD-binding motif [9]. However, the last residue was found to be glycine (G) in *Helicoverpa armigera*. The variations in this last residue could link to molecular functions of the conserved motifs, which are still not as clearly understood as the functions of the other two conserved residues (L and G).

Cluster analysis of the 150 amino acid patterns showed similar separation of the conserved FAD-binding region in LAAOs into two major clades as proposed by Hughes [11], the vertebrate-related clade and the mollusc-related clade, which also included fungi, bacteria, most actinopterygians, and one reptile. These clades were different from those found in the protein phylogenetic tree (Fig. 3), suggesting that these 47 amino acids were unequally affected by selection. Dividing them into sub-regions would allow better understanding of the functional conservation within this FAD-binding region. Amino acid variations or patterns within the first three sub-regions (bFAD, FAD, and FAD-GG), except those of the conserved residues, could undergo relaxed selection by allowing mutation of amino acids with similar biochemical properties because they did not affect the structural conformation of this conserved region, which was important to the co-factor-binding function of the LAAO enzyme. Variations in sequences and patterns within the GG and aGG sub-regions were more relaxed because these sub-regions might have evolved more flexibly to maintain their interactions with the FAD molecule and connect to the neighbouring part of the LAAO protein, which might have varied and diversified since the divergence from their most recent common ancestors. However, these 150 patterns of the FAD-binding region in the LAAO proteins were also different from those of other flavoproteins in the amine oxidase family. This conserved region of gastropods and other invertebrates was particularly unique, hence confirming that our designed *LAAO2* primers would amplify specifically the FAD-binding region of the *LAAO* gene in these organisms. The primers could be further used to amplify this *LAAO*-conserved region in a wide range of gastropods and other invertebrates.

Analysis of structural patterns also showed the separation of these conserved FAD-binding regions of LAAOs into two major clades, the mollusc-fungi-related clade and the vertebrate-related clade, similar to the phylogenetic trees and the clustering by amino acid patterns. This method showed some differences compared to the first clustering method. The patterns of bacteria and actinopterygians were moved to the vertebrate-related clade, and the close relationship of the conserved FAD-binding region of LAAOs in gastropods and fungi was also confirmed. Functional conservation could restrict dramatic change in these conserved structures of vertebrates. Outside the conserved region, more structural variations including the aGG sub-region, which was close to the other part of the enzyme, could allow diversification of the protein structures in different organisms. Thus, molecular phylogenetics and pattern analyses of sequence and structural variation could reflect evolutionary history and possible structure-function relationships of the conserved FAD-binding region of LAAOs in certain microorganisms, invertebrates and vertebrates.

In conclusion, this study has successfully designed and implemented two pattern analyses to complement phylogenetic data with the sequence-structure relationship of the conserved FAD-binding region of the LAAO enzyme for the first time. The findings will potentially pave the way to understand evolutionary history of the FAD-binding region of the *LAAO* gene in gastropods and other invertebrates. The strong positive selection on a few patterns of the conserved region in these invertebrates remains unanswered. Further study of other regions in the *LAAO* gene will help better understanding the biochemical reactions and support future investigation of novel LAAOs in gastropods and other invertebrates.

The following are the supplementary data related to this article.

Supplementary Table 1. Sample collection from different habitats. Supplementary Table 1

Supplementary Fig. 1. Patterns of the LAAO conserved domain (47 amino acids) from 231 samples (21 gastropods and 210 other organisms). Square boxes are coloured in pink for mammals, dark green for Aves, orange for reptiles, pale green for actinopterygians, dark purple for gastropods, pale purple for other invertebrates, blue for fungi, and yellow for bacteria. Amino acids are labelled with different colours according to their biochemical properties. Supplementary Fig. 1

Supplementary Fig. 2. Patterns of the before-FAD-binding-motif (bFAD) sub-region (5 amino acid residues) from 231 samples. Colour and label codes are the same as in Supplementary Fig. 1. Supplementary Fig. 2

Supplementary Fig. 3. Patterns of the FAD-binding-motif (FAD) sub-region (6 amino acid residues) from 231 samples. Colour and label codes are the same as in Supplementary Fig. 1. Supplementary Fig. 3

Supplementary Fig. 4. Patterns of the between-FAD-and-GG-motif (FAD-GG) sub-region (21 amino acids) from 231 samples. Colour and label codes are the same as in Supplementary Fig. 1. Supplementary Fig. 4

Supplementary Fig. 5. Patterns of the GG-motif sub-region (8 amino acids) from 231 samples. Colour and label codes are the same as in Supplementary Fig. 1. Supplementary Fig. 5

Supplementary Fig. 6. Patterns of the after-GG-motif (aGG) sub-region (7 amino acids) from 231 samples. Colour and label codes are the same as in Supplementary Fig. 1. Supplementary Fig. 6
