# Supplementary material for: Amplification and bioinformatics analysis of conserved FAD-binding region of L-amino acid oxidase (LAAO) genes in gastropods compared to other organisms
Source: Comput Struct Biotechnol J. 2018 Mar 2;16:98–107. doi: 10.1016/j.csbj.2018.02.008 (PMC6303269; doi:10.1016/j.csbj.2018.02.008)
Supplement: Supplementary Table 1. — Sample collection from different habitats. [file mmc1.doc]

**Appendix: Supplementary figures and tables**

**Supplementary Table 1** Sample collection from different habitats

| **Group** | **Species** | **Localities name** |
| --- | --- | --- |
| Gastropod | *Achatina fulica* | Backyard garden, Bangkok |
| Gastropod | *Cryptozona siamensis* | Tropical rain forest, Nakhon Ratchasima |
| Gastropod | *Semperula siamensis* | Backyard garden, Bangkok |
| Gastropod | *Physa* sp. | Freshwater ponds, Pathum Thani |
| Gastropod | *Lymnaea* sp. | Freshwater ponds, Nakhon Pathom |
| Gastropod | *Biomphalaria* sp. | Freshwater ponds, Bangkok |
| Gastropod | *Babylonia areolata* | Local market, Rayong |
| Gastropod | *Pomacea canaliculata* | Freshwater canal, Bangkok |
| Gastropod | *Viviparus* sp. | Freshwater ponds, Bangkok |
| Gastropod | *Bithynia* sp. | Freshwater ponds, Bangkok |
| Gastropod | *Melanoides* sp. | Freshwater ponds, Bangkok |
| Gastropod | *Cyclophorus volvulus* | Tropical rain forest, Nakhon Ratchasima |
| Gastropod | *Jorunna funebris* | Thai gulf, Chon Buri |
| Bivalve | *Hyriopsis bialata* | River, Ubonrachatani |
| Bivalve | *Perna* sp. | Local market, Samut Prakan |
| Bivalve | *Trisidos semitorta* | Local market, Rayong |
| Bivalve | *Anadara* sp. | Local market, Rayong |
| Bivalve | *Amusium pleurohectaus* | Local market, Rayong |
| Cephalopod | *Loligo* sp. | Local market, Rayong |
| Annelid | *Haemadipsa sylvestris* | Tropical rain forest, Chantaburi |
| Arthropod | *Portunus pelagious* | Andaman sea, Trang |
| Arthropod | *Helicoverpa armigera* | Farm, Bangkok |
