# Supplementary figures and images for: Amplification and bioinformatics analysis of conserved FAD-binding region of L-amino acid oxidase (LAAO) genes in gastropods compared to other organisms
Source: Comput Struct Biotechnol J. 2018 Mar 2;16:98–107. doi: 10.1016/j.csbj.2018.02.008 (PMC6303269; doi:10.1016/j.csbj.2018.02.008)

## Slide 1
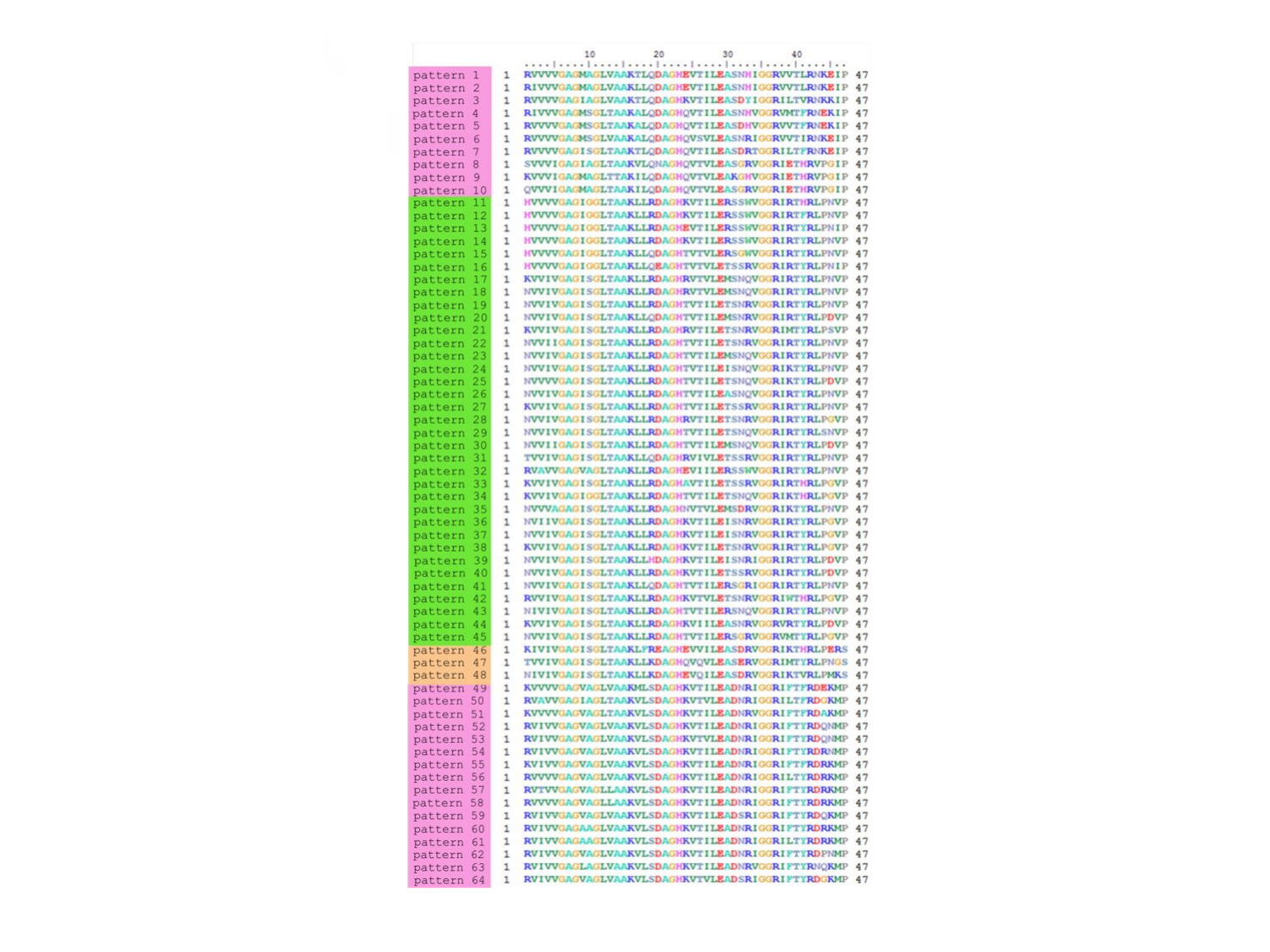

## Slide 2
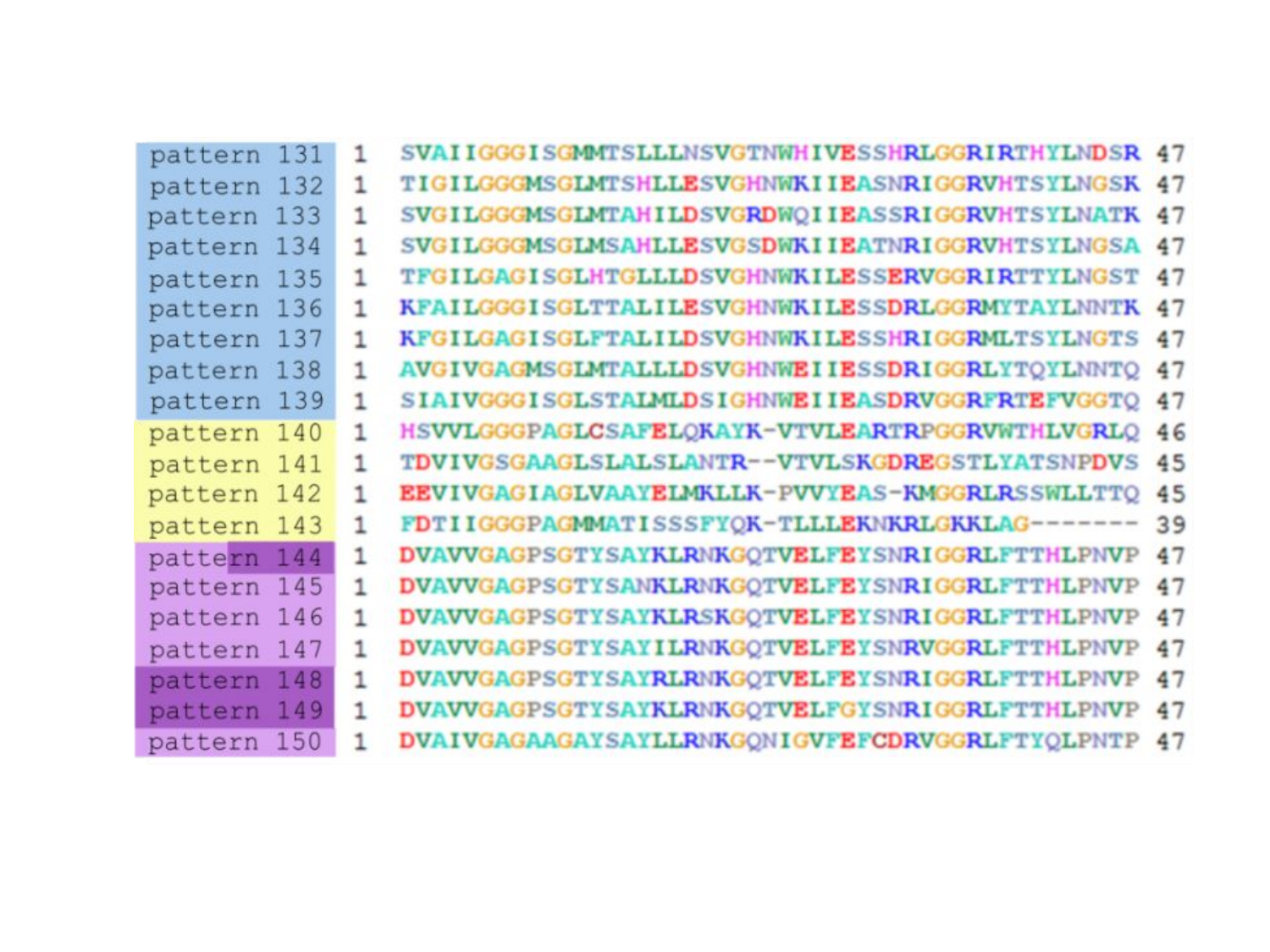

Supplement: Supplementary Fig. 1. — Patterns of the LAAO conserved domain (47 amino acids) from 231 samples (21 gastropods and 210 other organisms). Square boxes are coloured in pink for mammals, dark green for Aves, orange for reptiles, pale green for actinopterygians, dark purple for gastropods, pale purple for other invertebrates, blue for fungi, and yellow for bacteria. Amino acids are labelled with different colours according to their biochemical properties. [file mmc2.pptx]

## Slide 1
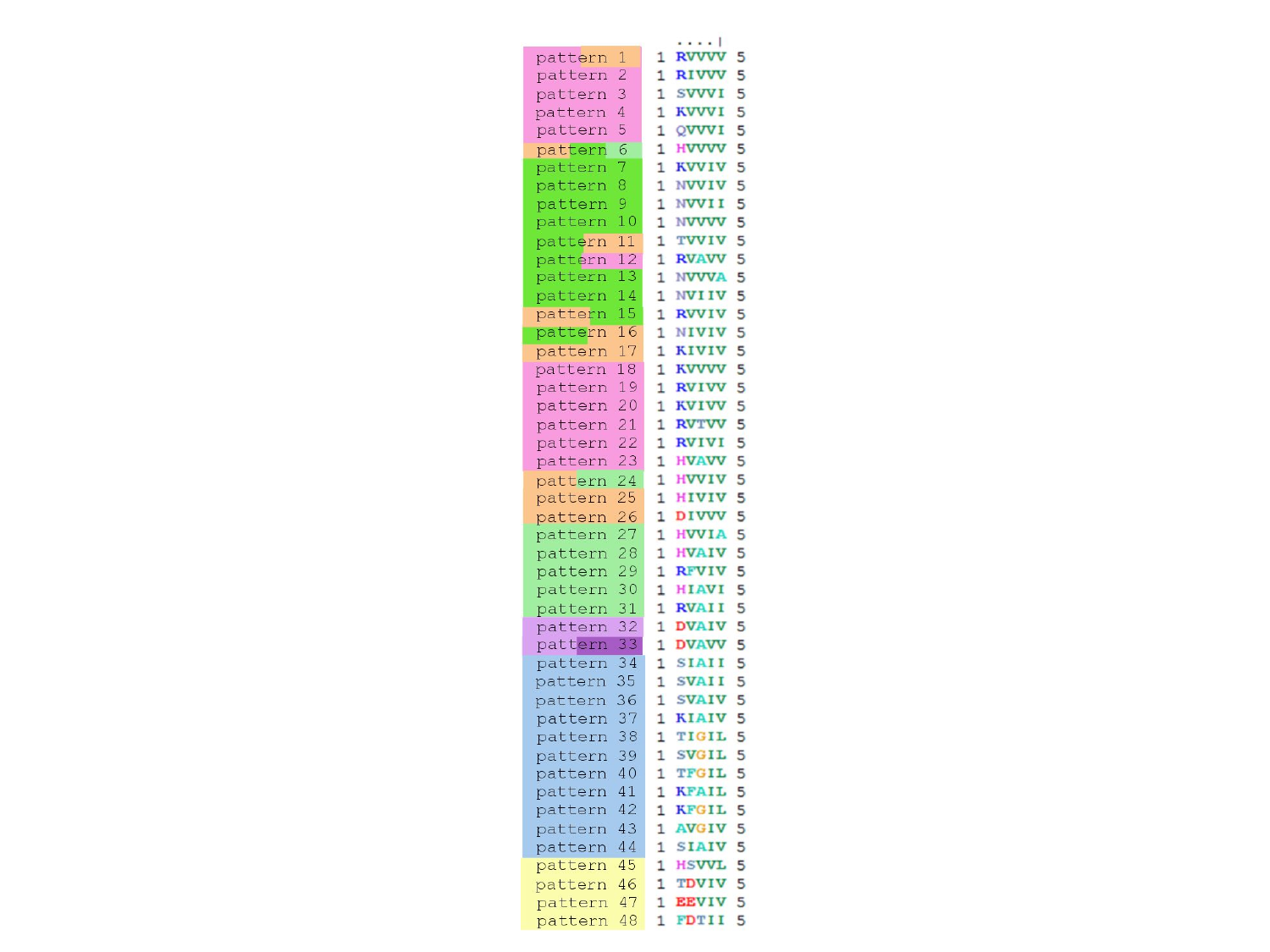

Supplement: Supplementary Fig. 2. — Patterns of the before-FAD-binding-motif (bFAD) sub-region (5 amino acid residues) from 231 samples. Colour and label codes are the same as in Supplementary Fig. 1. [file mmc3.pptx]

## Slide 1
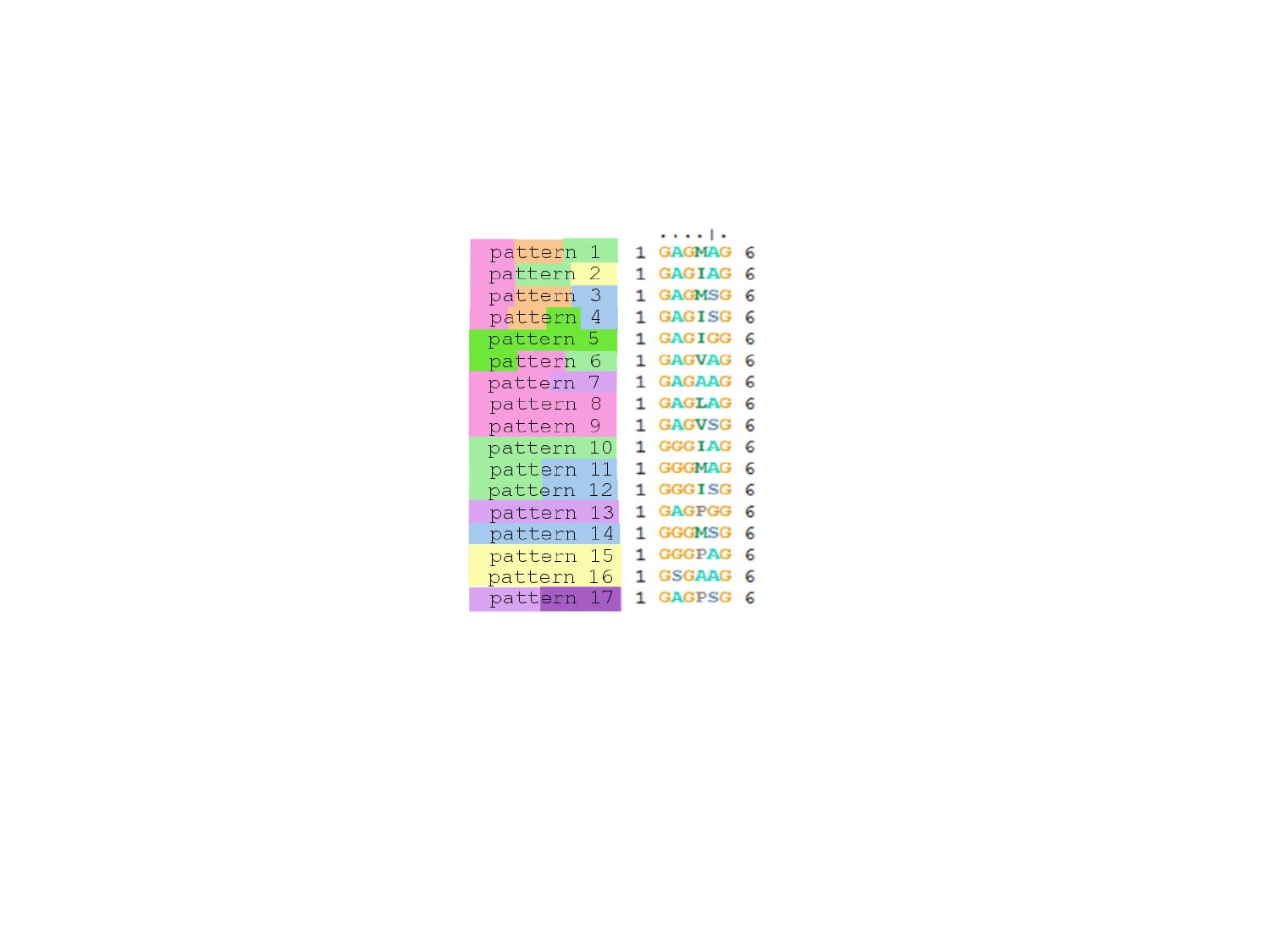

Supplement: Supplementary Fig. 3. — Patterns of the FAD-binding-motif (FAD) sub-region (6 amino acid residues) from 231 samples. Colour and label codes are the same as in Supplementary Fig. 1. [file mmc4.pptx]

## Slide 1
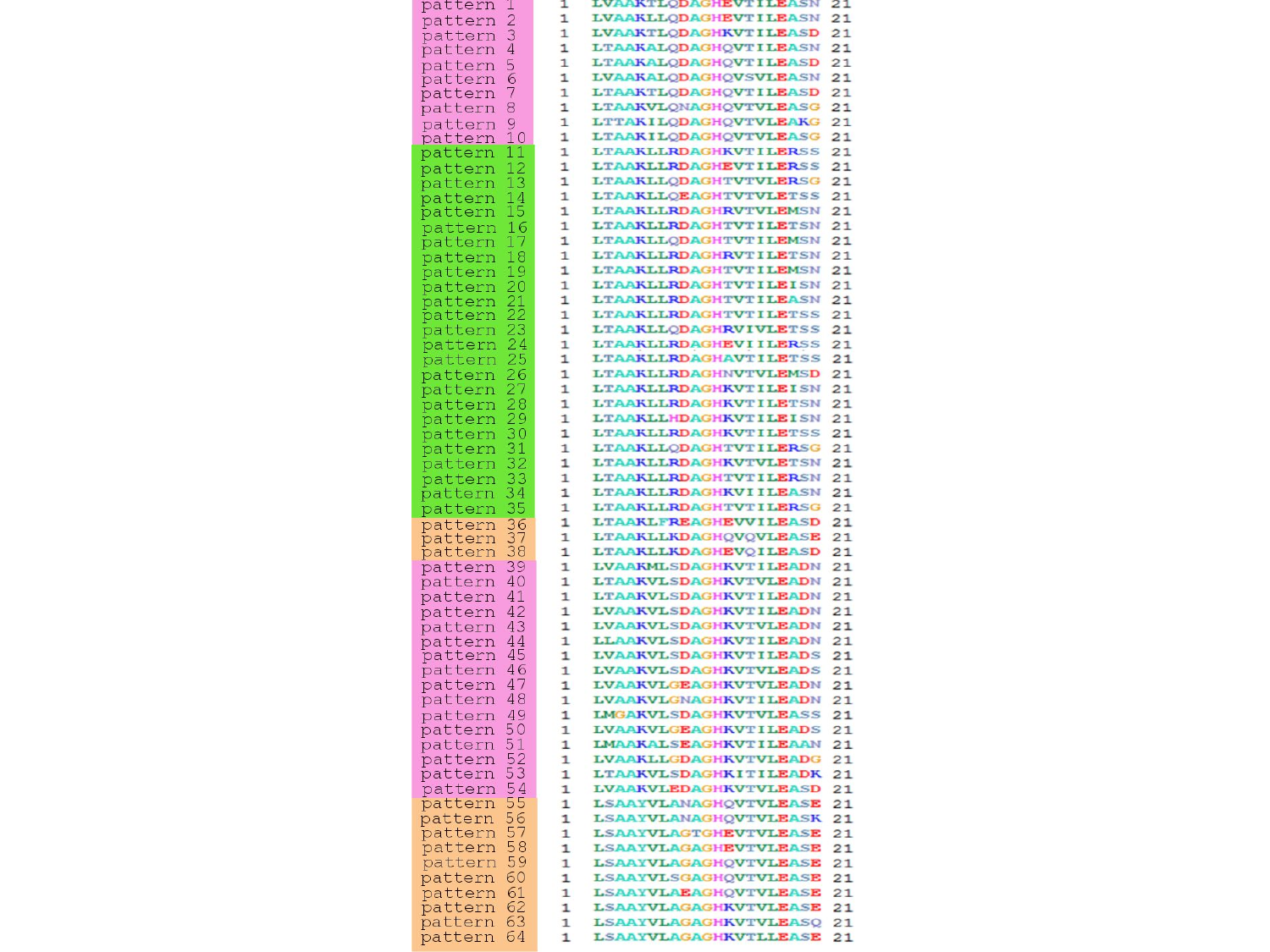

## Slide 2
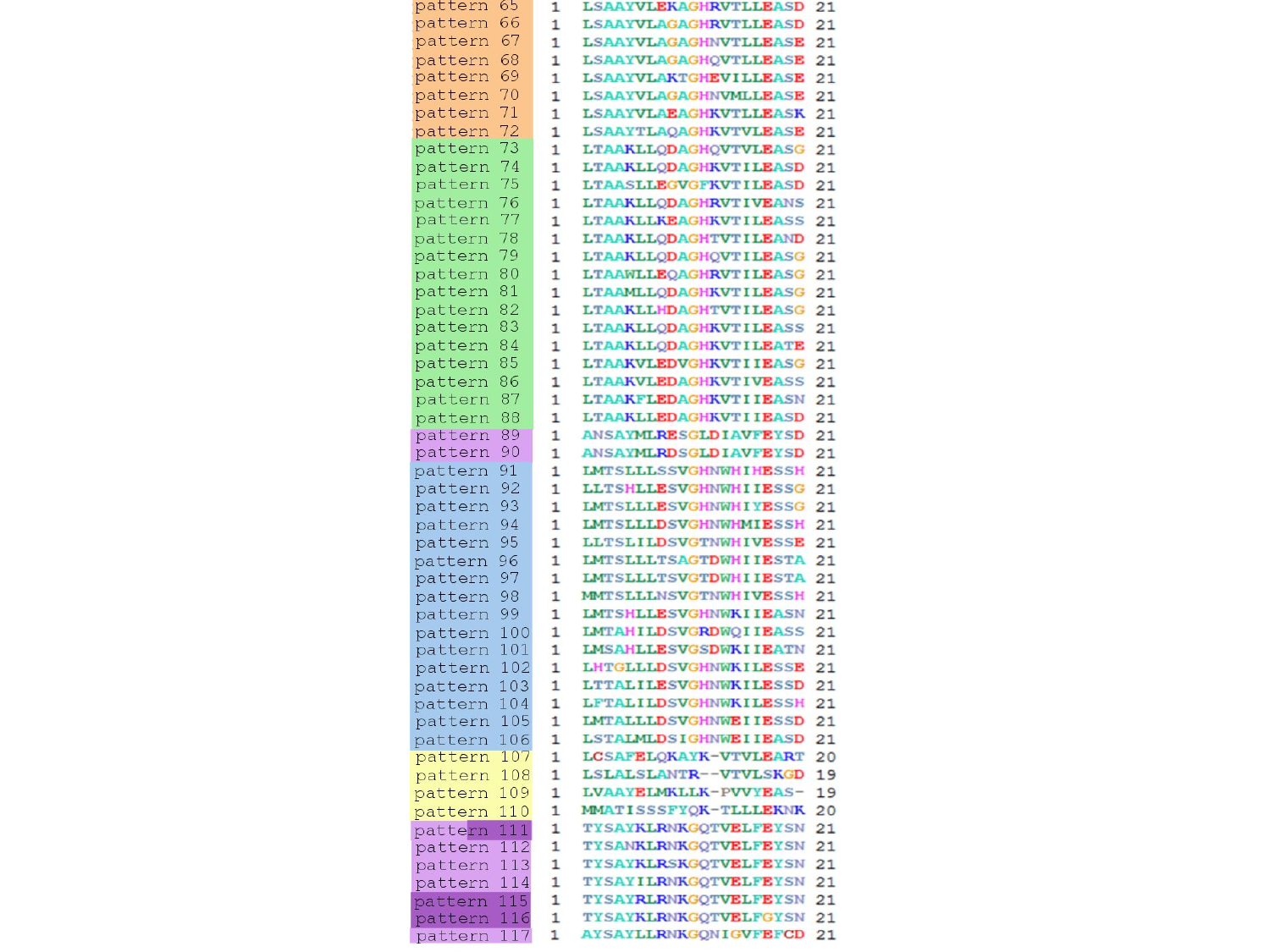

Supplement: Supplementary Fig. 4. — Patterns of the between-FAD-and-GG-motif (FAD-GG) sub-region (21 amino acids) from 231 samples. Colour and label codes are the same as in Supplementary Fig. 1. [file mmc5.pptx]

## Slide 1
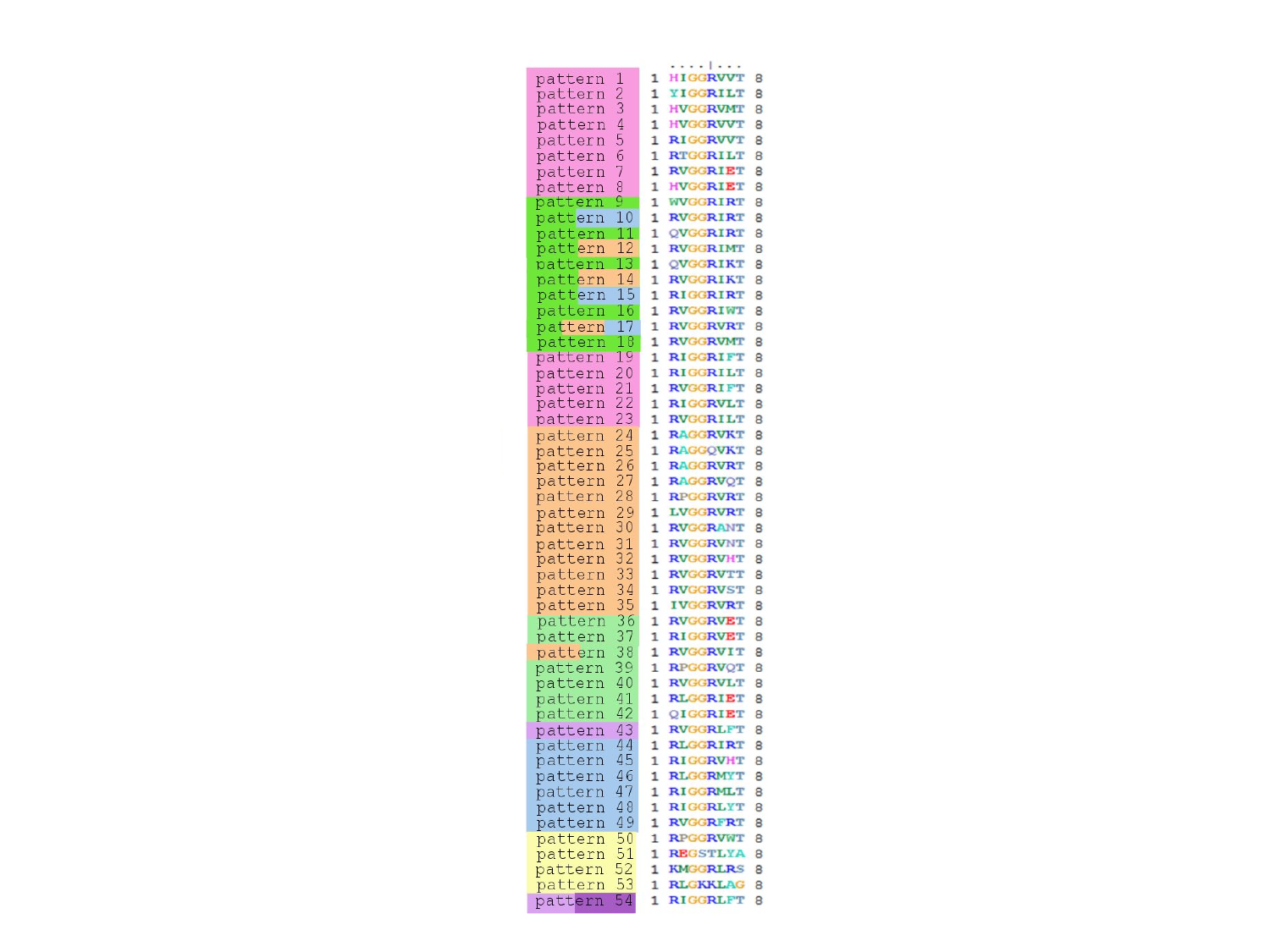

Supplement: Supplementary Fig. 5. — Patterns of the GG-motif sub-region (8 amino acids) from 231 samples. Colour and label codes are the same as in Supplementary Fig. 1. [file mmc6.pptx]

## Slide 1
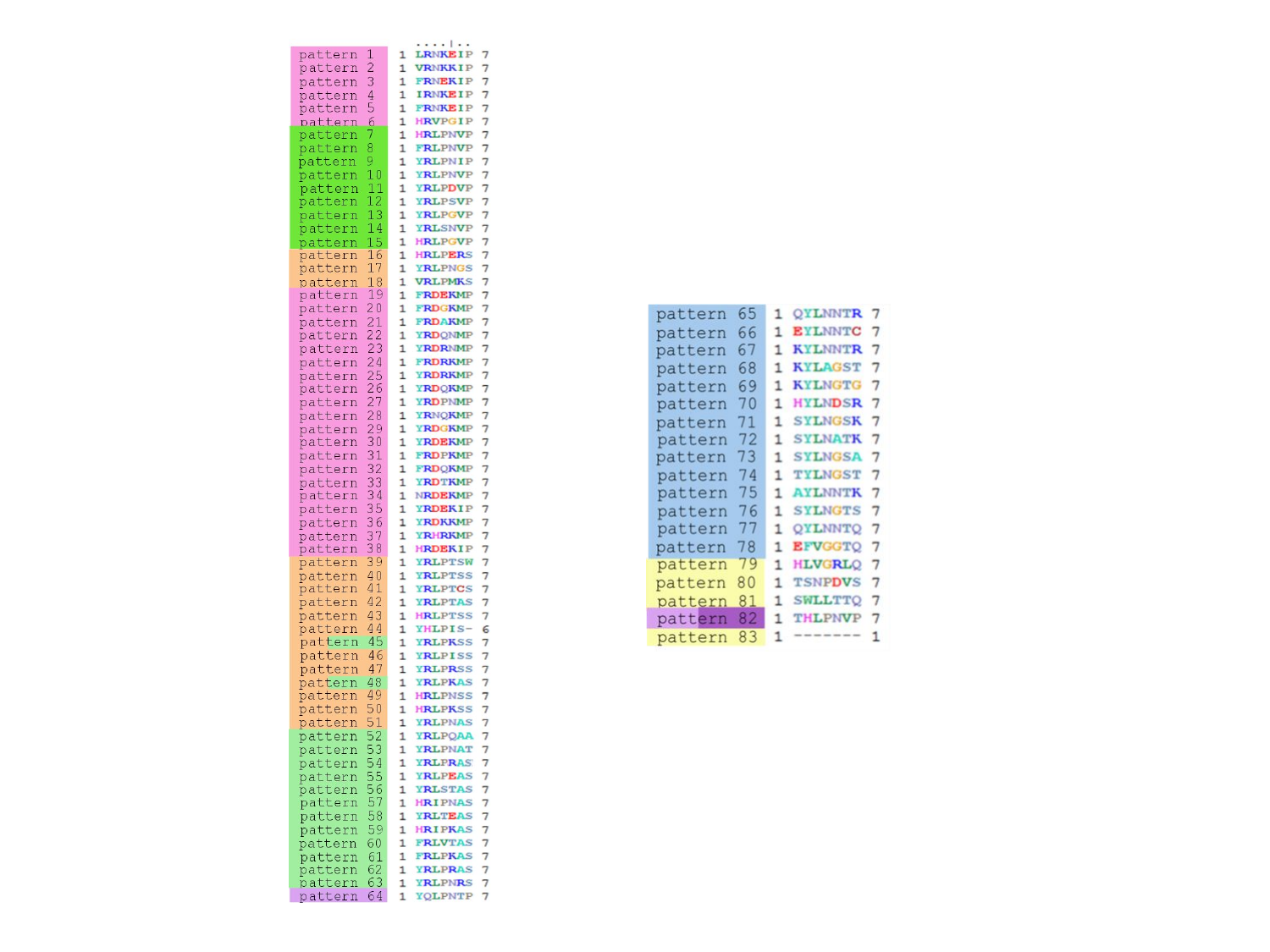

Supplement: Supplementary Fig. 6. — Patterns of the after-GG-motif (aGG) sub-region (7 amino acids) from 231 samples. Colour and label codes are the same as in Supplementary Fig. 1. [file mmc7.pptx]
